# MicroRNA-24 inhibits growth, induces apoptosis, and reverses radioresistance in laryngeal squamous cell carcinoma by targeting X-linked inhibitor of apoptosis protein

**DOI:** 10.1186/s12935-015-0217-x

**Published:** 2015-06-17

**Authors:** Li Xu, Zhifeng Chen, Fei Xue, Wei Chen, Ruina Ma, Shiyin Cheng, Pengcheng Cui

**Affiliations:** Department of Otolaryngology-Head and Neck Surgery, Tangdu Hospital and Laboratory for Laryngotracheal Reconstruction, Fourth Military Medical University, Xi’an, Shaanxi 710038 PR China; Department of Otolaryngology-Head and Neck Surgery, Nanjing General Hospital of Nanjing Military Command, Nanjing, Jiangsu 210002 PR China; Department of Otolaryngology-Head and Neck Surgery, Lanzhou General Hospital of Lanzhou Military Command, Lanzhou, Gansu 730050 PR China

**Keywords:** miR-24, Laryngeal squamous cell carcinoma, XIAP, Growth, Apoptosis

## Abstract

**Background:**

Increasing evidence indicates that dysregulation of microRNAs is involved in tumor progression and development. The aim of this study was to investigate the expression of microRNA-24 (miR-24) and its function in laryngeal squamous cell carcinoma (LSCC).

**Methods:**

Quantitative RT-PCR (qRT-PCR) was used to detect miR-24 expression in LSCC cell lines and tissue samples. MTT, colony formation, and flow cytometry was performed to analyze the effects of miR-24 expression on growth, apoptosis, and radiosensitivity of LSCC cells. Dual-luciferase reporter assays were performed to examine regulation of putative miR-24 targets. Expression of X-linked inhibitor of apoptosis protein (XIAP) mRNA and protein, cleaved or total caspase-3, and cleaved or total PARP protein were detected by qRT-PCR and western blotting assays, respectively.

**Results:**

miR-24 expression levels in LSCC cell lines or tissue were significantly lower than in a normal human keratinocyte cell line or adjacent normal tissues. Functional analyses indicated that re-expression of miR-24 inhibits growth, reduces colony formation, and enhances apoptosis in LSCC cells. In addition, miR-24 upregulation increases LSCC sensitivity to irradiation by enhancing irradiation-induced apoptosis, and luciferase activity indicated that miR-24 binds to the 3′-untranslated region (3′-UTR) of XIAP mRNA. Upregulation of miR-24 inhibits XIAP protein expression in LSCC cells, and silencing of XIAP mimics the effects of miR-24 upregulation on LSCC cells. In addition, XIAP mRNA expression significantly increases in LSCC tissues and is inversely correlated with miR-24 expression.

**Conclusions:**

Our data suggest that miR-24 inhibits growth, increases apoptosis, and enhances radiosensitivity in LSCC cells by targeting XIAP. Therefore, miR-24 may be a potential molecular target for the treatment of human LSCC.

## Introduction

Laryngeal squamous cell carcinoma (LSCC), the most common cancer of the upper digestive tract, accounts for approximately 14 % of head and neck squamous cell carcinoma (HNSCC) [1]. Recent advances in the multidisciplinary management of the early stages of the disease include surgical extirpation or larynx-preservation protocols using chemoradiotherapy, but a substantial proportion of patients with localized or locally advanced disease will eventually relapse and die [2]. Laryngeal carcinogenesis is a complex multistep process involving genetic dysregulation of proto-oncogenes and tumor suppressor genes [3]. Thus, it is imperative to understand the molecular mechanisms that underlie LSCC development, which may be helpful in identifying novel therapeutic targets.

MicroRNAs (miRNAs) are a class of small non-coding RNAs, 17–25 nucleotides long, that associate with 3′-untranslated regions (3′-UTR) of specific target messenger RNAs (mRNAs), targeting them for degradation or translational inhibition [4, 5]. Increasing evidence demonstrates that dysregulated miRNAs play critical roles in many biological processes, including growth, apoptosis, development, and tumorigenesis [6, 7]. Recently, the role of miRNAs in LSCC development has been the subject of several reports. Analysis of DNA microarray-based miRNA expression profiles derived from formalin-fixed paraffin-embedded (FFPE) tissue blocks of larynx LSCC, Li et al. observed differentially expressed miRNAs that may serve as potential molecular biomarkers for predicting metastatic development in LSCC [8]. By detecting these LSCC-specific miRNAs in plasma, Ayaz et al. identified differential expression of miRNAs in LSCC patient plasma, which may be potential early markers [9]. In addition, identification of miRNAs and mRNAs associated with multidrug resistance of human laryngeal cancer, suggest that miRNAs may be potential biomarkers for chemosensitivity prediction and drug resistance targets in LSCC [10]. These studies suggest that dysregulation of miRNAs may play important roles in LSCC progression and development. MiR-24 is an abundant miRNA encoded by the corresponding gene that maps to human chromosome 9q22 and 19p13 regions, which is well conserved between species and is expressed in normal tissues such as adipose, mammary gland, kidney, and differentiated skeletal muscle [11]. It has been reported that miR-24 functions as a tumor suppressor in a variety of human cancers, including tongue squamous cell carcinoma, osteosarcoma, bladder cancer, and gastric cancer [12–15]. However, the role of miR-24 in LSCC development and its possible molecular mechanisms are largely unclear and remain to be further elucidated.

In the present study, we show that miR-24 is downregulated in LSCC cell lines and tissue. Additional experiments demonstrate that re-expression of miR-24 inhibits growth, enhances apoptosis, and reverses chemoresistance in LSCC cells by directly targeting XIAP. These results suggest that targeting miR-24 may be a potential strategy for treating LSCC.

## Results

### MiR-24 expression is downregulated in LSCC cells and tissues

**Fig. 1:**
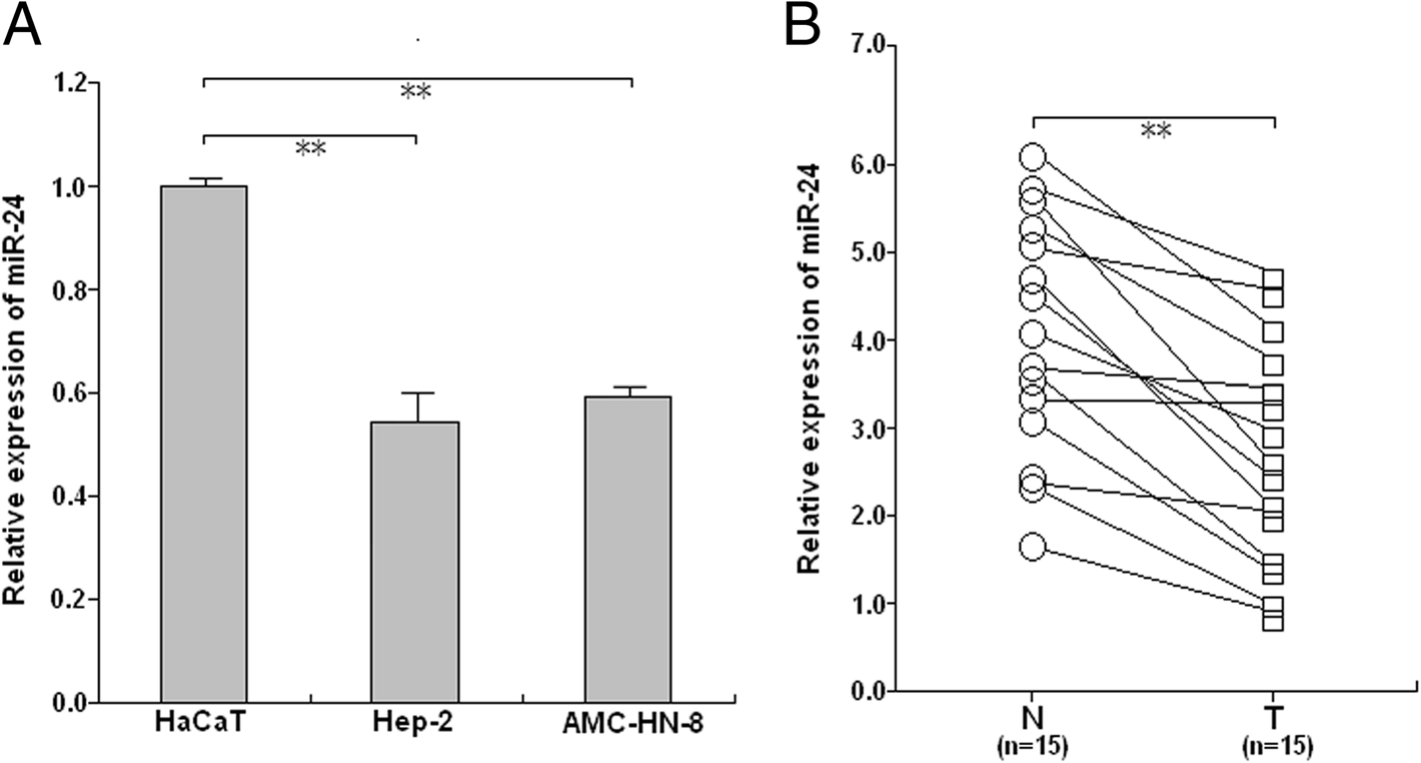
qRT-PCR of miR-24 expression in LSCC cells and tissue samples. **a** qRT-PCR detection of miR-24 expression in Hep-2 and AMC-HN-8 and HaCaT cells. **b** qRT-PCR detection of miR-24 expression in 15 paired LSCC and adjacent normal tissues. U6 was used as an internal control. Each assay was performed at least in triplicate. Corresponding *P* values determined by *t*-tests are indicated. T: LSCC tissues; N: adjacent normal tissues. **P < 0.01 *vs* control

Our qRT-PCR results indicate that miR-24 expression in Hep-2 and AMC-HN-8 was lower than that in HaCaT (*P* < 0.01, Fig. 1a). We also observed that miR-24 expression was significantly lower in LSCC tissues compared with that in the adjacent normal tissues (*P* < 0.01; Fig. 1b). These results indicate that downregulation of miR-24 may play an important role in LSCC development.

### Re-expression of miR-24 inhibits growth and enhances apoptosis in LSCC cells

**Fig. 2:**
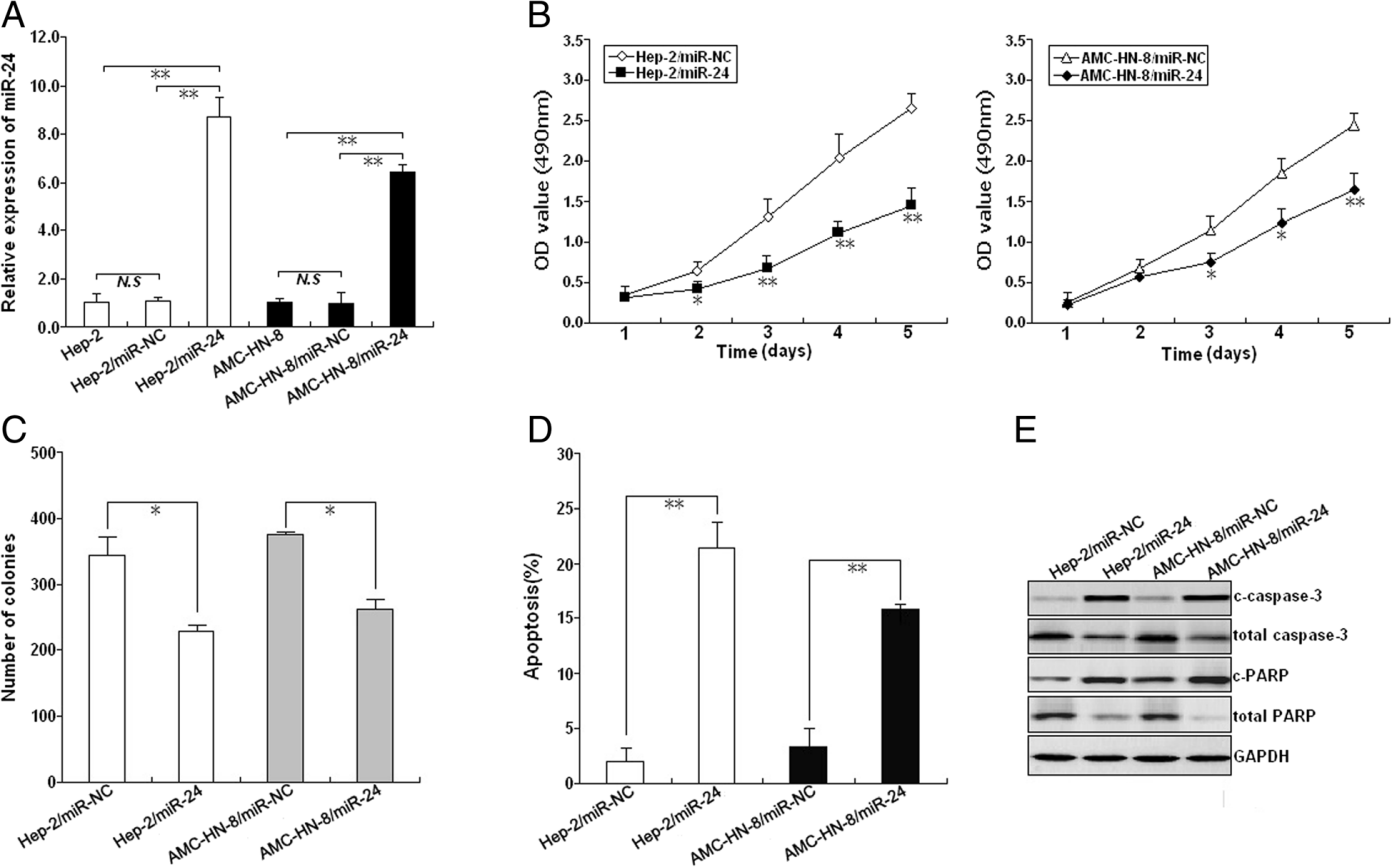
Effects of miR-24 expression on growth, colony formation, and apoptosis in LSCC. **a** qRT-PCR of miR-24 expression in mock or stably transfected Hep-2 and AMC-HN-8 cells. U6 was used as an internal control. **b** MTT analysis of Hep-2 and AMC-HN-8 growth following stable transfection with pGCMV/miR-NC or pGVMV/miR-24, respectively. **c** Colony formation assay was performed as described in Methods. **d** Flow cytometric analysis of apoptosis in Hep-2 and AMC-HN-8 stably transfected with pGCMV/miR-NC or pGVMV/miR-24, respectively. **e** Western blot detection of c-caspase-3, total caspase-3, c-PARP, and total PARP in the stably transfected Hep-2 and AMC-HN-8 cells. GAPDH was used as an internal control. Each experiment was performed at least in triplicate. *P < 0.05, **P < 0.01 *vs* control

To determine the effects of miR-24 expression on LSCC cells, pGCMV/miR-224 or pGCMV/miR-NC was stably transfected into Hep-2 and AMC-HN-8, and qRT-PCR used to confirm upregulation of miR-24 (Fig. 2a). The effects of miR-24 expression on LSCC growth were then examined by MTT and colony formation assays, indicating reduced growth (Fig. 2b). Similarly, colony formation capacity in Hep-2/miR-24 and AMC-HN-8/miR-24 cells was significantly reduced compared to controls (Fig. 2c). Flow cytometric analysis showed that miR-24 re-expression enhanced apoptosis in LSCC cells. Furthermore, upregulation of miR-24 increased expression levels of cleaved caspase-3 (c-caspase-3), cleaved PARP (c-PARP), and decreased expression levels of total caspase-3 and PARP (Fig. 2d). Therefore, re-expression of miR-24 appears to inhibit growth of LSCC cells by inducing caspase-3-dependent apoptosis.

### Re-expression of miR-24 increases radiosensitivity in LSCC cells by enhancing irradiation-induced apoptosis

**Fig. 3:**
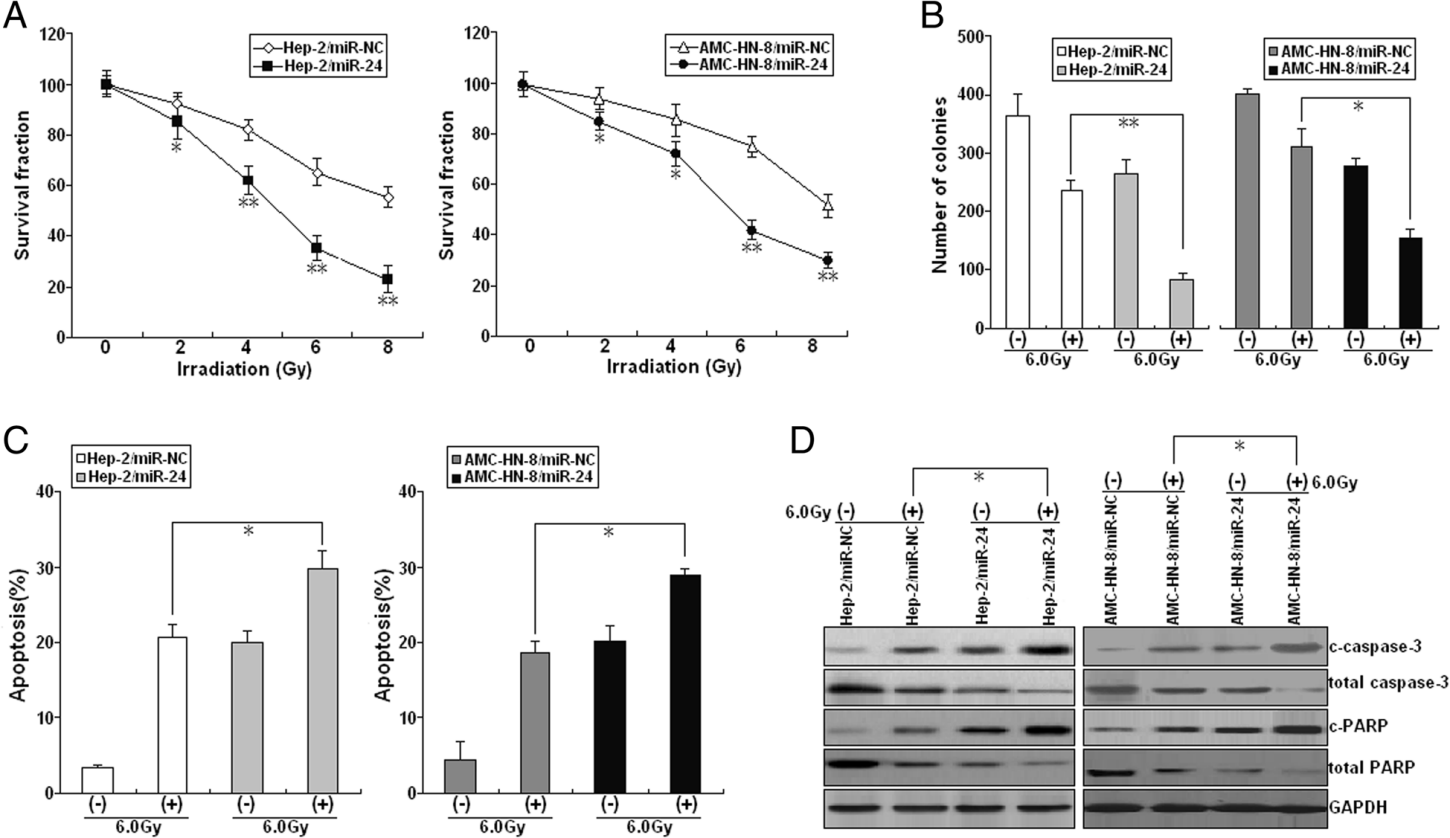
Effect of miR-24 expression on radiosensitivity of LSCC cells. **a** Radiosensitization by expression of miR-24 was evaluated based on clonogenic cell survival assays. Stably transfected Hep-2 and AMC-HN-8 cells were exposed to various doses of radiation prior to plating. **b** Colony formation assay was performed as described in Methods. **c** Flow cytometric detection of apoptosis in stably transfected Hep-2 and AMC-HN-8 cells with or without irradiation (6.0Gy). **d** Western blot detection of c-caspase-3, total caspase-3, cleaved c-PARP, and total PARP proteins in the stably transfected Hep-2 and AMC-HN-8 cells with or without irradiation (6.0Gy). GAPDH was used as an internal control. Each experiment was performed at least in triplicate. *P < 0.05, **P < 0.01 *vs* control

We sought to further examine the effects of miR-24 expression on radiosensitivity of LSCC. When combined with various doses of irradiation (0.0, 2.0, 4.0, 6.0 or 8.0 Gy), upregulation of miR-24 decreased growth of LSCC cells (Fig. 3a). Similarly, when combined with irradiation (6.0Gy), colony formation of Hep-2/miR-24 or AMC-HN-8/miR-24 cells was reduced in comparison with Hep-2/miR-NC or AMC-HN-8/miR-NC cells (Fig. 3b). Next, we analyzed the effects of miR-24 expression on apoptosis of LSCC cells following irradiation and found that miR-24 re-expression increased irradiation-induced apoptosis of LSCC cells (Fig. 3c). Upregulation of miR-24 increased expression of c-caspase-3 and c-PARP proteins in LSCC cells induced by irradiation treatment (Fig. 3d). Together, these data suggest that upregulation of miR-24 enhances LSCC irradiation sensitivity by increasing irradiation-induced apoptosis.

### XIAP as a direct target of miR-24 in LSCC cells

**Fig. 4:**
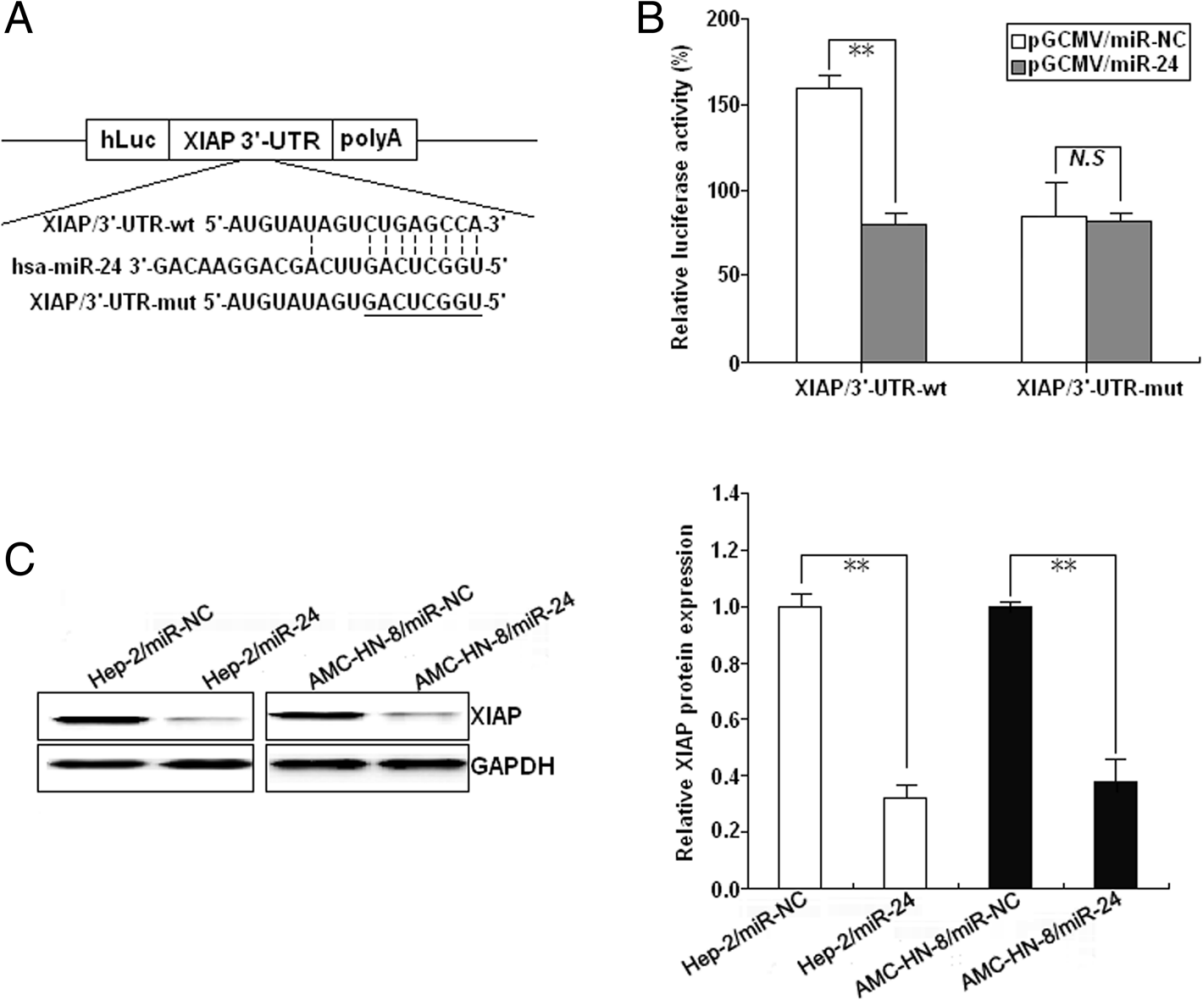
miR-24 binds to the 3′-UTR of XIAP mRNA. **a** A human XIAP/3′-UTR fragment containing wild-type or mutant miR-24-binding sequence was cloned downstream of the luciferase reporter gene in pLUC-luc. **b** pLUC-luc vector contains XIAP/3′-UTR-wt or XIAP/3′-UTR-mut and pGCMV/miR-24 or pGCMV/miR-NC were co-transfected into Hep-2 cells, and cell lysates prepared at 48 h for measuring luciferase activity, which was normalized to Renilla luciferase activity. **c** Western blot detection of XIAP protein expression in the stably transfected Hep-2 and AMC-HN-8 cells. GAPDH was used as an internal control. Each experiment was performed at least in triplicate. *P < 0.05, **P < 0.01 *vs* control

To explore how miR-24 affects malignant development of LSCC, we searched for potential regulatory targets of miR-24 using three prediction tools (miRanda, PicTar, and TargetScan), and identified a putative miR-24-binding site at positions 2301–2308 (CUGAGCCA) in the 3′-UTR of XIAP mRNA. To test directly whether XIAP is a target of miR-24, we constructed a luciferase reporter (pLUC/XIAP/3′-UTR-wt) in which the XIAP/3′-UTR nucleotides complementary to miR-24 (nt 2301–2308) were inserted into the pLUC vector. We also generated a mutant reporter (pLUC/XIAP/3′-UTR-mut), in which the first six nucleotides in the miR-24 seed region complementary sites were mutated (Fig. 4a). We then co-transfected appropriate plasmids with either the negative control miR-NC or miR-24 mimics into Hep-2 cells, and measured luciferase activity. Luciferase activity indicated that miR-24 inhibited signal compared with the miR-NC negative control (Fig. 4b), but had no effect on the activity of reporter vector containing the 3′-UTR of XIAP with six point mutations in the miR-24-binding site, suggesting that miR-24 interacts directly with the 3′-UTR of XIAP mRNA. Western blot results suggest that XIAP is controlled by miR-24 in Hep-2/miR-24 (or Hep-2/miR-NC) and AMC-HN-8/miR-24 (AMC-HN-8/miR-NC) cells, as miR-24 decreased expression of XIAP protein in LSCC cells (Fig. 4c). Collectively, these results indicate that XIAP may be a direct target of miR-24 in LSCC.

### Silencing of XIAP inhibits growth, increases apoptosis, and enhances radiosensitivity in LSCC cells

**Fig. 5:**
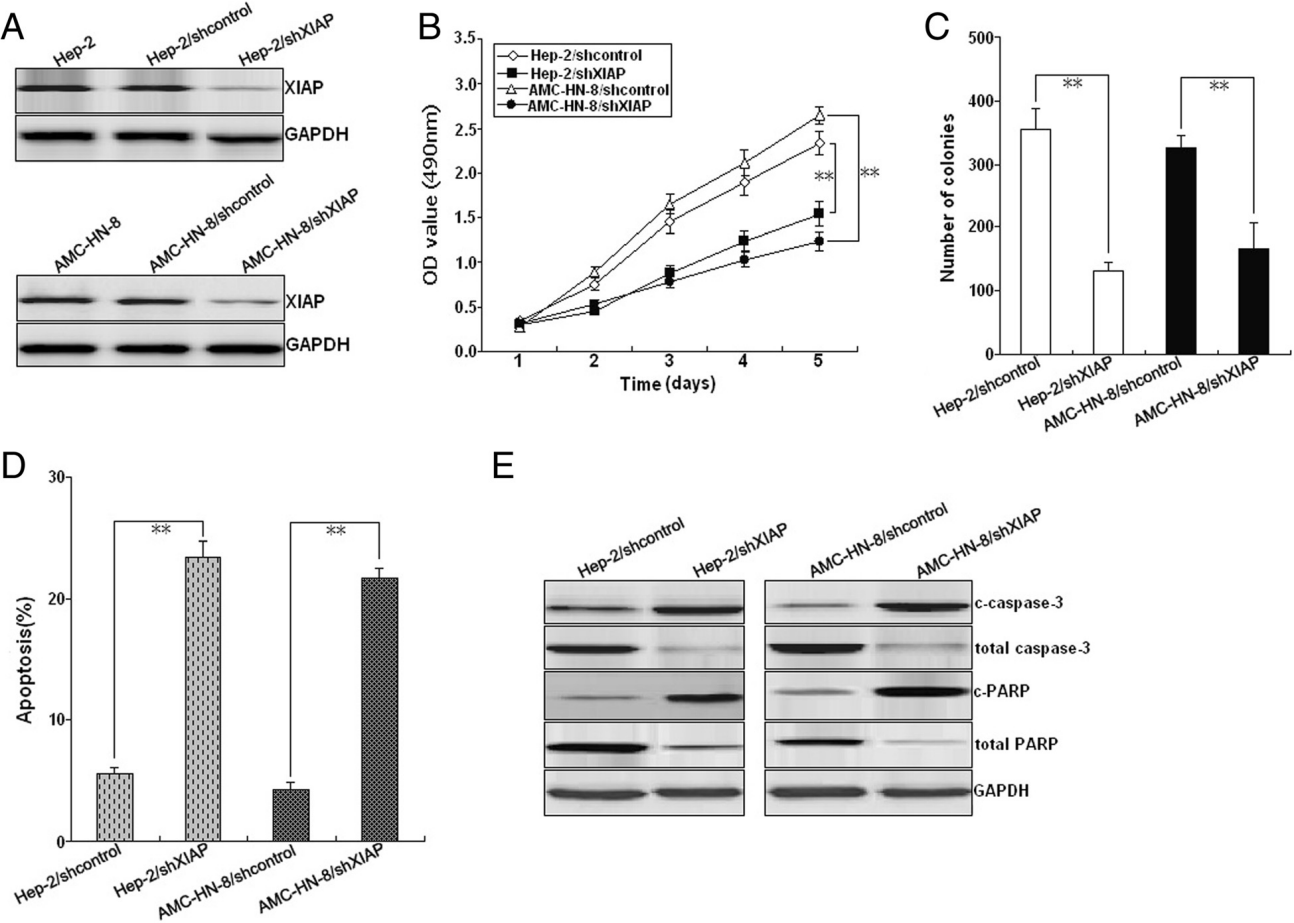
Effects of XIAP knockdown on growth, colony formation, and apoptosis in LSCC cells. **a** Western blot of XIAP protein expression in Hep-2 and AMC-HN-8 cells stably transfected with pSil/shXIAP or pSil/shcontrol, respectively. GAPDH was used as an internal control. **b** MTT analysis of growth in Hep-2 and AMC-HN-8 cells stably transfected with pSil/shXIAP or pSil/shcontrol, respectively. **c** Colony formation assay was performed as described in Methods. **d** Flow cytometric detection of apoptosis in Hep-2 and AMC-HN-8 cells stably transfected with pSil/shcontrol or pSil/shXIAP, respectively. **e** Western blot detection of c-caspase-3, total caspase-3, c-PARP, and total PARP proteins in the stably transfected Hep-2 and AMC-HN-8. GAPDH was used as an internal control. Each experiment was performed at least in triplicate. *P < 0.05, **P < 0.01 *vs* control

**Fig. 6:**
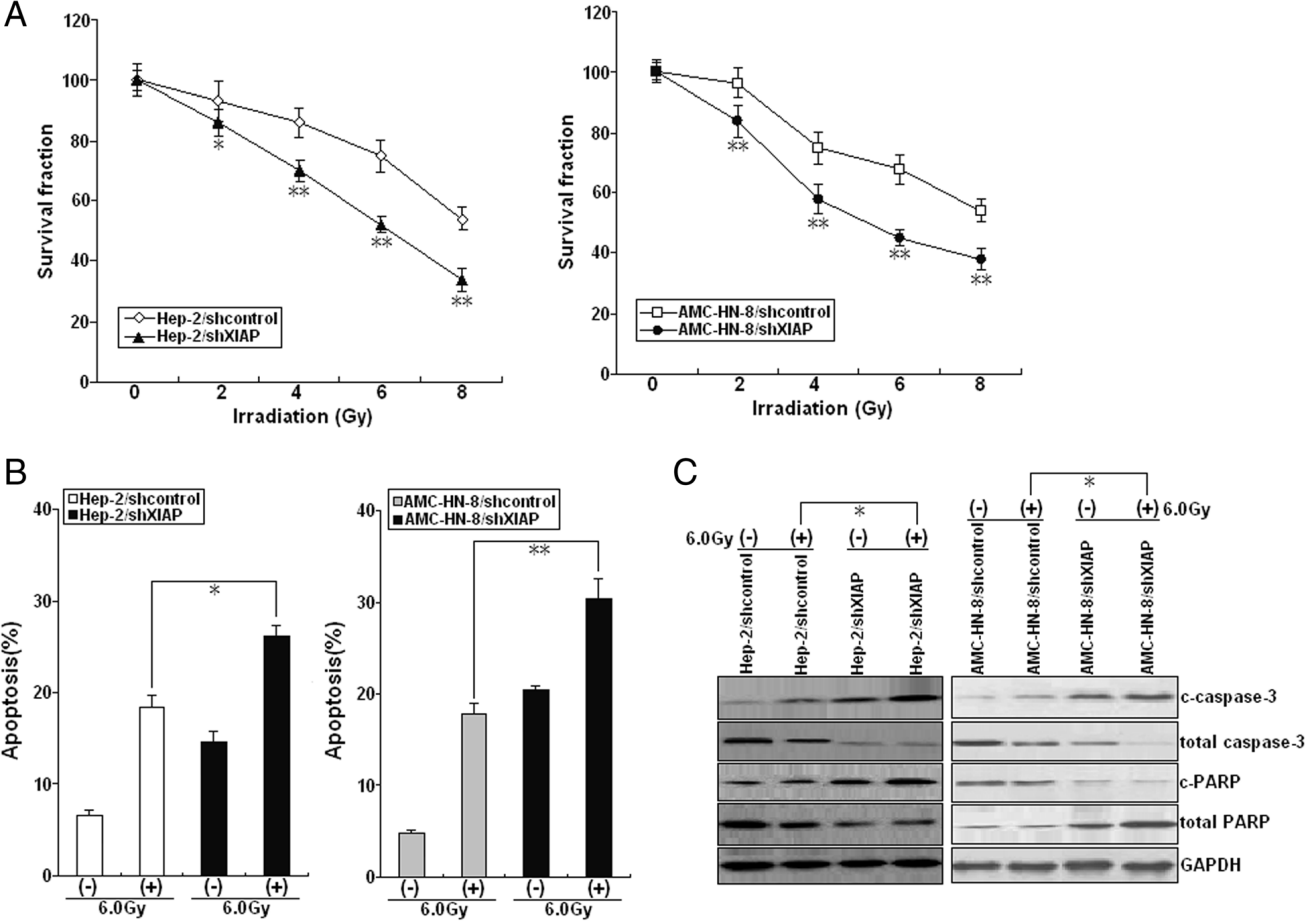
Effect of XIAP knockdown on LSCC radiosensitivity. **a** Radiosensitization by expression of XIAP was based on clonogenic cell survival assays. Stably transfected Hep-2 and AMC-HN-8 were exposed to various doses of radiation prior to plating for clonogenic cell survival. **b** Flow cytometry of apoptosis in stably transfected Hep-2 and AMC-HN-8 with (6.0Gy) or without irradiation. **c** Western blot of c-caspase-3, total caspase-3, cleaved c-PARP, and total PARP in stably transfected Hep-2 and AMC-HN-8 with or without irradiation (6.0Gy). GAPDH was used as an internal control. Each experiment was performed at least in triplicate. *P < 0.05, **P < 0.01 *vs* control

To validate that miR-24-mediated effects in LSCC cells resulted from targeting XIAP, pSil/shXIAP or pSil/shcontrol was stably transfected into Hep-2 (Hep-2/shXIAP or Hep-2/shcontrol) and AMC-HN-8 (AMC-HN-8/shXIAP or AMC-HN-8/shcontrol) cells. Western blot confirmed the decreased expression of XIAP protein in Hep-2/shXIAP or AMC-HN-8/shXIAP cells compared with mock Hep-2 or AMC-HN-8, and Hep-2/shcontrol or AMC-HN-8/sh controls (Fig. 5a). MTT and colony formation assays revealed that siRNA-mediated XIAP downregulation decreases growth and colony formation in LSCC cells (Fig. 5b, c). Flow cytometric analysis further indicated that silencing of XIAP increases caspase-3-dependent apoptosis in LSCC cells (Fig. 5d, e). Furthermore, silencing of XIAP increased the sensitivity of LSCC cells to irradiation by enhancing irradiation-induced caspase-3-dependent apoptosis (Fig. 6a-c). Therefore, silencing of XIAP mimics the effects of miR-24 upregulation on LSCC cells.

### XIAP is upregulated in LSCC tissues and is inversely correlated with miR-24 expression

**Fig. 7:**
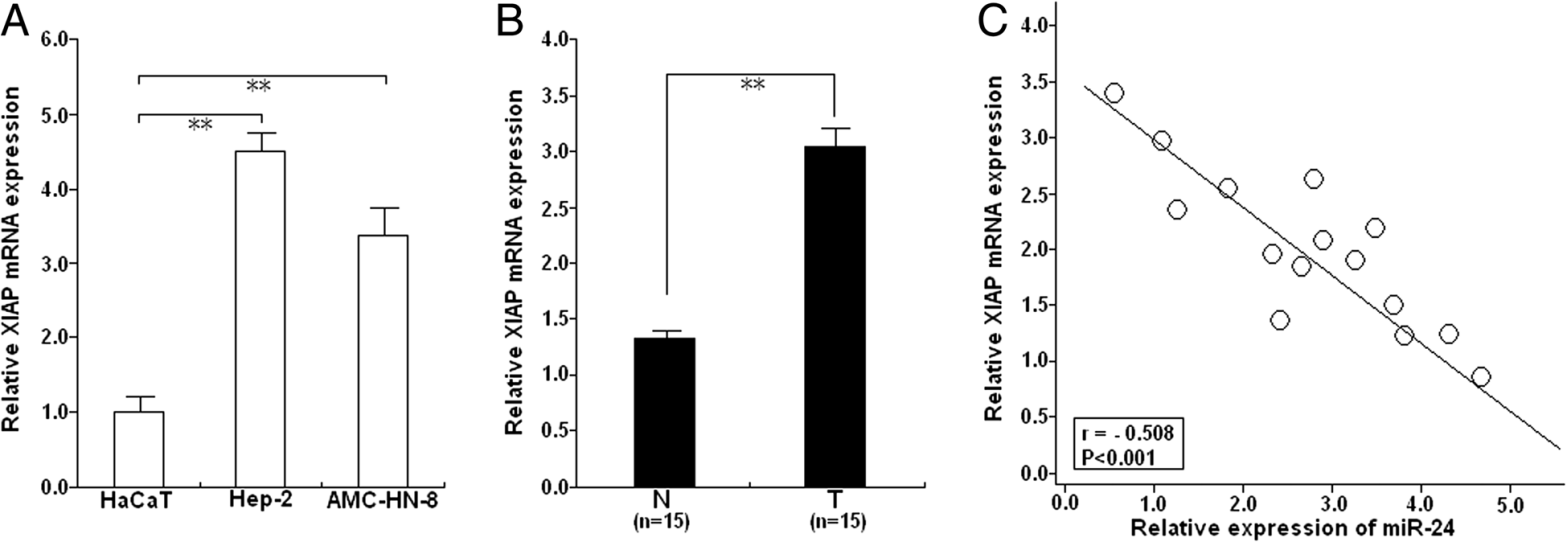
XIAP expression is upregulated in LSCC cells and tissues and inversely correlated with miR-24 expression. **a** qRT-PCR detection of XIAP mRNA expression in Hep-2 and AMC-HN-8, and HaCaT cells. GAPDH was used as an internal control. **b** qRT-PCR detection of XIAP mRNA expression in 15 paired LSCC tissues and adjacent normal tissues. GAPDH was used as an internal control. T: LSCC tissues; N: the adjacent normal tissues. **c** Statistical analysis reveals an inverse correlation between relative miR-24 and XIAP mRNA expression level in LSCC tissues (n = 15; r = −0.508; *P* < 0.001). Corresponding *P* values analyzed by Spearman correlation test are indicated. **P < 0.01 *vs* control

We further examined expression of XIAP mRNA in HaCaT and Hep-2 and AMC-HN-8 cells by qRT-PCR, and found that the expression of XIAP mRNA in HaCaT cell line was lower than that in the LSCC cell lines (Fig. 7a). In addition, expression of XIAP mRNA in 15 paired LSCC and adjacent normal tissues showed that XIAP mRNA in LSCC tissues was elevated (*P* < 0.001; Fig. 7b). We then observed that expression level of XIAP mRNA expression levels inversely correlated with the expression level of miR-24 in LSCC tissues (Pearson’s correlation, r = −0.508; *P* < 0.001; Fig. 7c). Therefore, the increased XIAP mRNA expression in LSCC tissues correlates with low-level miR-24 expression.

## Discussion

In the present study, we showed that miR-24 is downregulated in LSCC cell lines and tissues. Re-expression of miR-24 inhibited growth, enhanced apoptosis, and increased radiosensitivity in LSCC cells. Furthermore, XIAP, a member of the inhibitor of apoptosis family of proteins, was identified as a functional and direct target of miR-24. To the best of our knowledge, this is the first report to elucidate a role for miR-24 in LSCC, suggesting that reduced miR-24 plays a critical role in LSCC progression by inducing XIAP expression.

miRNAs, which are typically 18–22 nucleotides long, evolutionarily conserved single-stranded RNA molecules, regulate target genes by binding to complementary sequences in the 3′-UTR and play a role in many human physiological and pathological processes, including cancer [16, 17]. Recently, correlation of dysregulated miRNAs with LSCC has been reported. Using microarrays, Cao et al. identified 29 differentially expressed miRNAs between LSCC and adjacent normal tissues. They observed upregulation of miR-21, miR-93, miR-205, and miR-708, and downregulation of miR-125b and miR-145 [18]. In addition, through analysis of LSCC patient plasma miRNA, Ayaz et al. reported upregulated of 17 miRNAs whereas nine were downregulated. Importantly, five of these (miR-331-3p, 603, 1303, 660-5p, and 212-3p) are LSCC specific, suggesting that LSCC-specific plasma miRNAs might serve as novel noninvasive LSCC biomarkers [9]. In addition, miRNAs are correlated with growth, invasion, and metastasis of LSCC. For example, Zhao et al. showed that overexpression of miR-155 promotes proliferation and invasion of human LSCC by targeting SOCS1 and STAT3 [19]. Zhang et al. reported that down-regulation of miR-206 promotes proliferation and invasion of laryngeal cancer via VEGF expression [20]. Furthermore, Tian et al. also showed that miR-203 is downregulated in LSCC and suppresses proliferation and induces tumor apoptosis [21]. Additional dysregulated miRNAs contribute to LSCC progression, such as miR-93, miR-27a, and miR-375 [22–24]. These reports suggest that targeting dysregulated miRNAs might be a potential strategy for the treatment of human LSCC. Recently, miR-24 was shown to be downregulated in nasopharyngeal carcinoma (NPC) and that is inhibits NPC cell growth, promotes apoptosis, and suppresses the growth of NPC xenografts [25]. However, miR-24 expression and its effects on LSCC are unclear. Here, we show that miR-24 expression in LSCC cell lines was significantly lower than that in a human keratinocyte cell line. In addition, miR-24 expression in LSCC tissues was downregulated in comparison with that in adjacent normal tissues. Functional analyses demonstrated that re-expression of miR-24 could inhibit growth, reduce colony formation, and enhance caspase-3-dependent apoptosis in LSCC cells. Radiotherapy is a crucial treatment in the management of LSCC, but little tumor-controlling efficacy in some cases is achieved because of inherent radioresistance. Therefore, a better understanding of molecular mechanisms in LSCC radioresistance may help identify a novel molecular target for radiosensitization of LSCC. Here, we investigated the effects of miR-24 expression on radiosensitivity of LSCC, with the results suggesting that miR-24 re-expression increased the sensitivity of LSCC to irradiation by enhancing irradiation-induced apoptosis. Thus, restoration of miR-24 may be a better method for reversing radioresistance in LSCC.

miRNAs exert their function in a dynamic and context-dependent manner by targeting diverse downstream target genes. Thus, identification of target genes may elucidate miR-24 function and the molecular mechanisms by which it mediates LSCC progression. XIAP was identified as a direct target mRNA of miR-24 by bioinformatics analysis. Previously, Xie et al. reported that miR-24 regulates XIAP to reduce the apoptosis threshold in cancer cells [26]. However, whether miR-24 targets XIAP to affect LSCC is poorly understood. Human XIAP, which belongs to the inhibitor of apoptosis (IAP) family, is an inhibitor of apoptotic cell death that protects cells by caspase-dependent and independent mechanisms [27]. This protein is overexpressed in many human cancers, which may lead to formation of malignant phenotypes, such as growth, apoptosis, and chemo and radioresistance. For example, Zhang et al. reported that transfer of siRNA against XIAP induces apoptosis and reduces tumor cell growth in human breast cancer in vitro and in vivo [28]. Shrikhande et al. showed that silencing XIAP decreases gemcitabine resistance of pancreatic cancer cells [29]. In LSCC, Wang et al. showed that RNA interference-mediated downregulation of XIAP expression inhibits proliferation and induces apoptosis but also diminishes LSCC radioresistance [30]. However, whether miRNAs play a critical role in activation of XIAP in LSCC requires further investigation, although luciferase assays indicated that miR-24 binds to the 3′-UTR of XIAP mRNA. In concordance with luciferase reporter results, overexpression of miR-24 downregulates endogenous XIAP protein levels in LSCC. Furthermore, LSCC XIAP mRNA expression correlated with miR-24 expression, and XIAP expression was elevated relative to that in a normal human keratinocyte cell line. Furthermore, miR-24 expression in LSCC tissues was higher than that in adjacent normal tissues, with XIAP expression inversely correlated with miR-24 expression. More importantly, small interfering RNA (siRNA)-mediated XIAP knockdown mimics the same effects of miR-24 upregulation on growth, apoptosis, and radiosensitivity in LSCC. These results suggest that XIAP mRNA may be a direct target of miR-24 in LSCC.

In conclusion, our study demonstrates that miR-24 is downregulated in LSCC and re-expression of miR-24 inhibits growth, enhances apoptosis, and increases radiosensitivity in LSCC. The data also demonstrate a functional link between miR-24 and XIAP in LSCC, suggesting that XIAP may be a target of miR-24, and that abnormal miR-24 and XIAP expression may be correlated with aggressive progression of LSCC. Several limits of our study should be noted. First, other miR-24 target mRNAs need to be identified. Second, a larger scale study with appropriate tissue samples is needed to investigate the clinicopathological and prognostic significance of miR-24. Third, only one tumor cell line was used in this study, so a larger number of tumor cell lines could be included in the future in terms of the fact that these cancers are heterogenous in nature.

## Materials and methods

### Tissue samples

Fifteen paired LSCC and adjacent normal tissues were collected from the Department of Pathology in Jingling Hospital between 2011 and 2013, after informed consent had been obtained. The ethics committee of Jiangsu Province Medical Association approved the study protocol. LSCC diagnosis was determined according to the latest WHO criteria and TNM stage classification (UICC 2002). None of the patients had received chemotherapy or radiotherapy before surgery. All tissue samples were snap-frozen in liquid nitrogen, and transferred to 500 ml TRIzol solution (Invitrogen, Carlsbad, CA, USA) immediately after harvesting to avoid mRNA degradation. Samples were stored in a biobank at −80 °C until processed.

### Cell culture

Two LSCC cell lines (Hep-2 and AMC-HN-8) and a normal human keratinocyte cell line (HaCaT) were purchased from Shanghai Institute Chinese Academy of Science (Shanghai, China), cultured in RPMI 1640 media (Invitrogen) supplemented with 10 % fetal bovine serum (FBS), and 100 μM each of penicillin and streptomycin in a humidified atmosphere of 5 % CO_2_ at 37 °C.

### Transfection of plasmids

For ectopic expression of miR-24 or knockdown of XIAP, pGCMV/miR-24 (or pGCMV/miR-NC vector) or pSil/shXIAP (pSil/shcontrol) were purchased from GenePharm (Shanghai, China). Transfections were performed using Lipofectamine™ 2000 (Invitrogen) according to the manufacturer’s instructions. Cells were transfected with recombinant DNA vectors containing a G418 selection marker and selected on G418 (Sigma, St. Louis, MO, USA) at 600 mg/ml for 4 weeks. Single clones were maintained in G418 at 100 mg/ml.

### qRT-PCR assay

Total RNA was isolated using Trizol (Invitrogen), and 10 μg RNA used to synthesize cDNA with Super-Script II First-Strand Synthesis System (Invitrogen), or TaqMan® MicroRNA Reverse Transcription Kit (Applied Biosystems). Aliquots of the reaction mixture were used for real-time PCR with Power SYBR Green PCR Master Mix or with the TaqMan® 2 × Universal PCR Master Mix. PCR reaction conditions: 50 °C for 20 s, 95 °C for 10 min followed by 40 cycles of 95 °C for 15 s, 60 °C for 1 min. We calculated a △Ct (target-reference), which is equal to the difference between threshold cycles for miR-24 (target) and the threshold cycle for U6 RNA (reference) (△Ct (target-reference) = Ct target-Ct reference). The fold-change between patient or cell sample and a normal control for miR-24 or XIAP was calculated by the 2ˉ^ΔΔCt^ method.

### Western blotting assay

Cells were lysed using the mammalian protein extraction reagent RIPA (Beyotime, Beijing, China) supplemented with a protease inhibitor cocktail (Roche, Pleasanton, CA, USA), and phenylmethylsulfonyl fluoride (PMSF) (Roche). Approximately 50 μg protein extract was separated by 10 % SDS-PAGE, transferred to 0.22 μm nitrocellulose (NC) (Sigma), and incubated with specific antibodies. Autoradiograms were quantified by densitometry using Quantity One software (Bio-Rad, Brea, CA, USA). Rabbit anti-XIAP, cleaved caspase-3, total caspase-3, cleaved PARP, and total PARP were obtained from Cell Signaling Technology (Danvers, MA, USA). GAPDH antibody was used as a control.

### Cell growth assay

Cell growth was measured by MTT assay (Sigma). In brief, cells were seeded into five 96-well culture plates with each plate having all three kinds of cells (6-parallel wells/group). Each day, 200 μL MTT (5 mg/mL) was added to each well, and the cells incubated at 37 °C for 4 h. Reactions were stopped by lysing cells with 150 μL DMSO for 5 min. Optical density was determined on a Versamax microplate reader (Molecular Devices, Sunnyvale, CA, USA) at 490 nm.

### Colony formation assay

Cells were trypsinized to single cell suspensions and seeded to 6-well plates at 600/well. After 14 days culture in RPMI 1640 medium, colonies were stained with Giemsa solution and the number of colonies counted. Each experiment was performed in triplicate.

### Flow cytometric detection of apoptosis

The cells were harvested, washed twice with cold PBS, fixed in ice-cold 70 % ethanol, and incubated overnight at −20 °C. Cells were then stained with 40 μg/mL of propidium iodide (PI) for 30 min. A minimum of 1.0 × 10^6^ cells were collected and analyzed using Cell Quest software (Becton Dickinson Co., Franklin Lakes, NJ, USA), and the percentage of cells with apoptotic nuclei (% apoptosis) calculated.

### Clonogenic survival assay

Cells were seeded into 6-well plates and cultured overnight, radiated (0.0, 2.0, 4.0, 6.0 and 8.0 Gy), and cultured for another 14 days. Colonies (>50 cells) were fixed with chilled methanol and stained with crystal violet, and counted on an inverted microscope. The surviving fraction was calculated as follows: number of colonies/number of plated cells. All the procedures were repeated in triplicate.

### Dual-luciferase reporter assay

To validate XIAP as a direct target of miR-24, we performed dual-luciferase reporter assays using a pLUC target reporter plasmid containing XIAP/3′-UTR (pLUC/XIAP/3′-UTR-wt). Additionally, we generated a mutant XIAP/3′-UTR reporter construct by site-directed mutagenesis of the putative miR-24 target site in wild-type XIAP/3′-UTR (pLUC/XIAP/3′-UTR-mut) using Stratagene QuikChange® Site-Directed Mutagenesis Kit (Stratagene, Heidelberg, Germany). Cells were transiently cotransfected for 24 h with reporter plasmids (200 ng) and pGCMV/miR-24 (or pGCMV/miR-NC) and harvested in reporter lysis buffer. Both firefly and Renilla luciferase activities were measured using the Dual-Luciferase assay kit (Promega, Madison, WI, USA) according to the manufacturer’s instructions. Luciferase activity was normalized against protein concentration and expressed as a ratio of firefly to Renilla luciferase unit.

### Statistical analysis

Statistical analysis was performed with SPSS version 13.0 (SPSS Inc., Chicago, IL, USA). Values are expressed as the mean ± SD. *χ*^2^ test and *t*-test were applied as appropriate. Correlations were evaluated by Spearman’s rank correlation coefficients. A *P* < 0.05 was considered statistically significant.

## Abbreviations

miRNA: microRNA
NC: Negative control
qRT-PCR: Quantitative real-time polymerase chain reaction
LSCC: Laryngeal squamous cell carcinoma
3′-UTR: 3′-untranslational region
wt: Wild type
mut: Mutant type
PI: Propidium iodide
siRNA: Small interfering RNA
XIAP: X-linked inhibitor of apoptosis protein
